# When Should a Patient with Statin-Induced Myopathy Be Re-challenged? A Case of Necrotizing Autoimmune Myopathy

**DOI:** 10.1155/2018/1215653

**Published:** 2018-10-01

**Authors:** Elena Obreja, Pamela Sequeira, Diana Girnita

**Affiliations:** ^1^Internal Medicine Resident, Weiss Memorial Hospital, Chicago, IL, USA; ^2^Department of Pathology, Trihealth Hospitals, Cincinnati, OH, USA; ^3^Department of Rheumatology, Trihealth Physician Partners, Cincinnati, OH, USA

## Abstract

Statins are notorious for causing myalgia and sometimes mild elevation of CPK (creatine phosphokinase). Herein, we present a case of necrotizing autoimmune myopathy induced by statins. The patient was on therapy with atorvastatin for about six years before she started developing myalgia and mild elevation in CPK that resolved after discontinuation of therapy. Since her cardiovascular risk was high and she had hypercholesterolemia, three months after CPK levels normalization, she was re-challenged with pravastatin. Few months later, she again presented severe myalgia, weakness, and elevated CPK levels. Hence, medication was discontinued, and she undergone an extensive workup for possible causes of inflammatory myopathies that revealed necrotizing autoimmune myopathy. Our case report offers an excellent source of “identification patterns” of muscular autoimmune disease which can be easily mistaken as common side effect of a drug.

## 1. Introduction

Statin-associated myopathy or myalgia is a well-known side effect of lipid-lowering agents. Usually, after discontinuation of the drug, symptoms alleviate and patients may be re-challenged with another statin. However, in rare cases, symptoms of myalgia do not subside after statins are discontinued. Therefore, we are presenting a case report of a rare autoimmune condition that can be induced by statins. We will present the importance of recognition of the clinical manifestations and laboratory changes that can lead to a correct diagnosis. The ultimate educational value of our case report is to highlight the approach in treating these patients and establish when and/or if is safer to expose them to other types of statins.

Necrotizing autoimmune myopathy (NAM) is distinguished from the other inflammatory processes by the acute onset of proximal muscles weakness associated with very high (more than 50 times the upper limit of normal) CPK levels [1]. Therefore, clinical picture, CPK levels, EMG, and MRI muscle are important steps in evaluating a patient with myalgia. However, when suspicion remains, only the muscle biopsy will be the gold standard in clarifying the diagnosis.

## 2. Case Presentation

**Figure 1:**
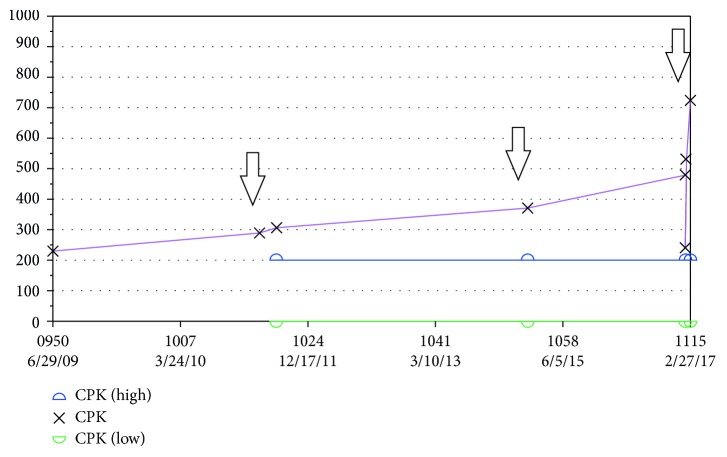
CPK levels during statin therapy, before presenting to Rheumatology Clinic.

A 67-year-old African American female with a past medical history significant for type 2 insulin-dependent diabetes mellitus, hypertension, hypercholesterolemia, severe osteoarthritis of the left shoulder, moderate degenerative disc disease of lumbar spine, gout, chronic kidney disease stage III, and chronic pancreatitis was referred to rheumatology with complains of weakness in her upper extremities proximal muscles for about one month. She was on treatment with atorvastatin for hypercholesterolemia for the last 6 years. Due to concern of statin-induced myopathy, the atorvastatin was discontinued a month before her consultation. Around the time of stopping atorvastatin, the patient described flu-like symptoms (low-grade fevers, myalgia, arthralgia, and runny nose). While she was on therapy with atorvastatin, baseline CPK levels were in the 230 mg/dl, with the highest value being 529 mg/dl at the time of therapy discontinuation (Figure 1).

Her CPK was repeated 20 days later and was increased to 720 mg/dl. The patient was seen in the rheumatology clinic within one week. At the time of her initial evaluation, the patient's main complaint was pain in the right shoulder, irradiating to her neck, right wrist, and fingers. Despite her subjective weakness, strength was 5/5 in her proximal and distal upper extremities muscles. She occasionally reported difficulties swallowing and photosensitivity, but denied any lower extremity weakness or difficulties to rise from a chair, rashes, oral/nasal ulcerations, Raynaud's phenomenon, or shortness of breath.

Her physical exam was suggestive of right shoulder impingement syndrome (significantly decreased range of motion, positive Neer's and Hawkins tests, anteroflexion 90°, reduced abduction, adduction, internal rotation, and external rotation), and right wrist examination was significant for mild swelling, limited range of motion, and tenderness to palpation. Left shoulder and wrist examination were unremarkable. Neck flexors and extensors examination was normal. Lower extremities examination revealed 5/5 strength; the patient was able to get out of the chair without pushing herself out.

Routine laboratory studies were significant for normocytic anemia; moderate elevated BUN, CRP of 2.5 mg/L, and CPK levels of 720 mg/dl.

Plain X-rays were obtained, and they were suggestive of severe degenerative osteoarthritis of the right shoulder, chondrocalcinosis of right wrist and knee, and diffuse osteopenia.

Due to her history of gout and CKD, an initial diagnosis of possible crystal induced arthropathy was made.

The patient received a steroid injection in the right shoulder and was started on a short taper of prednisone with complete resolution in her symptoms in two weeks.

At two-month follow-up, she was free of symptoms and CPK levels were normal (145 mg/dl).

Cardiovascular risk being high and having high cholesterol levels, the decision to re-challenge the patient with another statin was made. This time she was resumed on pravastatin, 3 months after her CPK levels were persistently normal, and she was free of symptoms.

**Figure 2:**
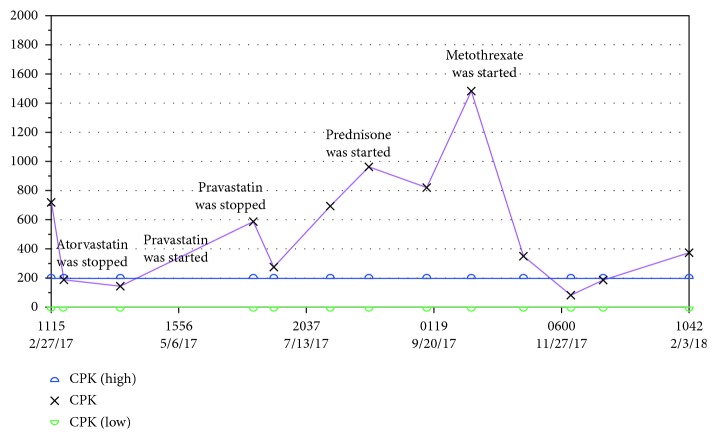
CPK levels after statin re-challenge and initiation of immunosuppressive therapy.

After three months on therapy with pravastatin, the patient experienced recurrent myalgia in proximal muscles of upper but also lower extremities. CPK levels increased again to 586 mg/dl, and the sedimentation rate (ESR) was 51 mm/hr (Figure 2). The pravastatin was discontinued.

A myositis panel was obtained and was negative for all antibodies (Table 1). Anti-HMG-CoA reductase antibody was not tested because the patient could not afford the cost of the test.

Therefore, an EMG was performed and revealed peripheral sensory neuropathy but no signs of myopathy. MRI of the right humerus was obtained and showed small bursal effusion, severe osteoarthritis, rotator cuff tear, severe chondral loss, severe tendinosis of subscapularis tendon, and full-thickness tear supraspinatus tendon, but no muscle edema. The patient was referred to neurology to evaluate for muscle weakness.

**Figure 3:**
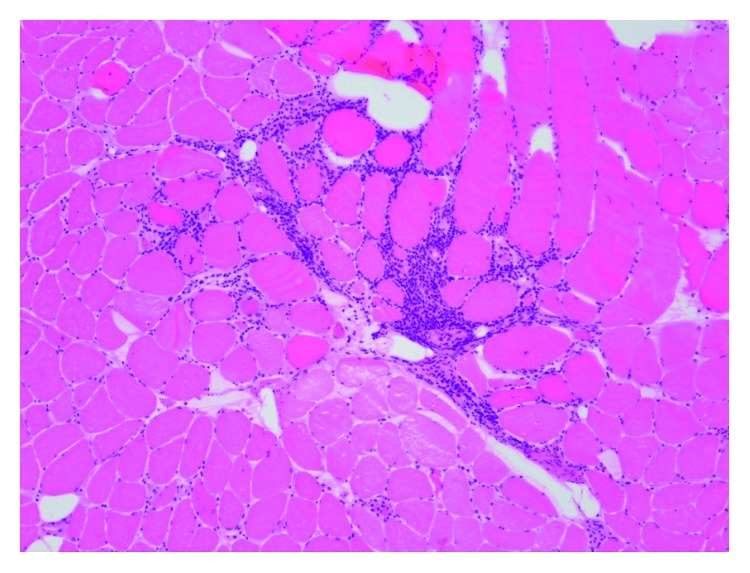
Muscle intrafascicular and perivascular lymphocytic inflammation (H&E, photomicrograph ×100).

**Figure 4:**
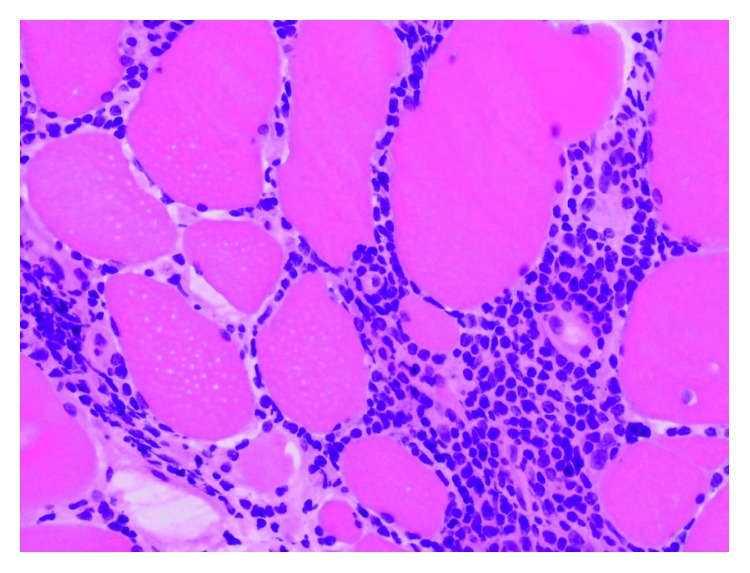
Lymphocytic inflammation surrounding muscle fibers (H&E, photomicrograph ×400).

Since her presentation was not consistent with a neurological disease and her CPK increased further to 1400 mg/dl, we made the decision to obtain muscle biopsy. The pathology report was consistent with inflammatory necrotizing myopathy (intrafascicular inflammation with muscle atrophy, the inflammation is predominantly intrafascicular with actively necrotic muscle fibers) (Figures 3 and 4).

## 3. Treatment

Prednisone 1 mg/kg/day was initiated with minimal improvement in her symptoms; however; CPK levels started to trend down, but did not normalize after one month on this therapy. Methotrexate was added as a steroid-sparing agent and gradually increased to 20 mg/week over the course of next 2 months. The prednisone was not able to be tapered down due to persistent severe myalgia in her upper and lower extremities as well as elevated levels of CPK (960 IU/L). Intravenous immunoglobulin (IVIG) therapy was initiated (dose: 1 gram for two consecutive days). In one month after first IVIG infusion, CPK levels normalized and the patient experienced significant clinical improvement. The therapy with prednisone, methotrexate, and IVIG will continue.

## 4. Discussion

Assessing serum creatine kinase (CPK) is an important step in evaluating a patient with complains of myalgia or weakness. CPK levels vary according to race, gender, age, and muscle mass. Higher levels were found in men and African Americans. A study done by National Health and Nutrition Examination Survey supported by CDC suggests that the racial differences are not explained by muscle mass but due to differential production or clearance of CPK [2]. The higher median CPK levels in African Americans men are 135 U/L versus 73 U/L in African American females, 64 U/L in white men versus 42 U/L in white women, and 69 U/L in Hispanic men versus 48 U/L in Hispanic women [3]. Therefore, being African American, our patient was at high risk to have higher CPK levels than median.

Among the clues that can lead us to the cause of myalgia associated with elevated CPK is carefully reviewing the medications patients are taking, especially lipid-lowering medication such as statins. Lipid-lowering agents act by inhibiting HMG-CoA reductase, thus reducing cholesterol biosynthesis. Usually statins are well tolerated and less than 0.5% of the patients' associated clinically significant myonecrosis are manifested by weakness and elevated CPK levels. Statin-associated muscle toxicity includes a wide spectrum of manifestations from simple myalgia (characterized only by muscle pain) to myopathy or myositis (which also associates elevated CPK levels more than 10 times the upper normal limit). Mild muscular symptoms such as myalgia occur in equal percent of patients treated with statins and placebo [4]. In all these cases, symptoms subside after statins are discontinued.

In our case, the initial diagnosis was statin-induced myopathy, so we discontinued statins which resulted in improvement of symptoms and normalization of CPK. However, in the patient's need of lipid-lowering medication, the decision was made to start her on a hydrophilic statin, like pravastatin. The sensitivity to one statin versus another is based on their lipophilic, like atorvastatin, or hydrophilic properties. Lipophilic statins are more toxic than the hydrophilic ones (rosuvastatin and pravastatin). It is explained by the capacity of the hydrophilic statins to be actively transported in the hepatocytes by expressing organic anionic transporting polypeptide 1B1 which regulates hepatic uptake. Thus, lipophilic statins diffuse into nonhepatocyte cells such as myocytes [5]. Furthermore, pravastatin is one of the statins that are not metabolized by CYP3A4 [6]. These mechanisms explain the statin-induced myopathy.

In our case, after re-exposing the patient to a “safer” statin, her myalgia got worse and CPK levels were found to be very high. Therefore, there was a suspicion of inflammatory myopathy and a more detailed workup was done.

Differentiating between the five types of inflammatory myopathies requires a stepwise approach that includes checking the levels of CPK, obtaining EMG, followed by MRI and ultimately performing a muscle biopsy.

In contrast to myalgia or myopathy, autoimmune necrotizing myopathy (NAM) is a very rare condition that is not always associated with statin use, and it accounts for 19% of all inflammatory myopathies [1]. Statin-associated NAM may also present with dysphagia, arthralgias, or Raynaud's phenomena [7]. There are several other causes of NAM besides the cholesterol-lowering drugs, such as paraneoplastic syndromes (most common types of cancer associated are lung, renal, breast, and ovarian cancer), connective tissue diseases, and HIV infection [8].

EMG in NAM is characterized only by active myopathic units, and the muscle biopsy shows the presence of necrotic fibers, regenerating fibers without significant inflammatory cells, and diffuse or focal upregulation of MHC class I expression [1]. Assessment of autoantibodies can also be helpful but anti-HMG-CoA reductase antibodies are not sensitive and may be present in patients that have not been exposed to statins [1, 9].

An importance in the diagnosis of NAM is identification of anti-signal recognition particle autoantibody (SRP) and 3-hydroxy-3-methylglutaryl-CoA-reductase (HMGCR) autoantibody [10]. These antibodies have been identified in 60% of the patients diagnosed with NAM [10]. A study done by Allenbach et al. reported a more severe disease in anti-SRP-positive patients versus anti-HMGCR-positive patients [11]. However, latest reports raise the question that these antibodies might have a possible protective role rather than pathogenic. SRP and HMGCR are involved in proteins synthesis, which can thus explain a regenerating process of the myofibers rather than destruction [10]. Either way, these antibodies can be useful for diagnosing NAM but are not the fundamental piece for diagnosis.

We could only assess the myositis antibodies for our patient since she could not afford to pay for anti-HMG-CoA reductase antibody testing.

A study in 2010 showed that, from all inflammatory muscle disorders, 82% of the patients with necrotizing myopathy have been previously exposed to statins as compared to the patient with dermatomyositis (18%), polymyositis (24%), and inclusion body myositis (38%) [12].

Cancer screening was also performed in our case. Colonoscopy, mammogram, and CT chest/abdomen/pelvis were all negative.

The mechanism by which statins can cause autoimmune necrotizing myopathy is supposed to be due to overexpression of HMG-CoA reductase in the majority of patients with class II HLA allele DRB1*∗*11 : 01 [9]. Studies have shown that people with this allele develop anti-HMG-CoA reductase antibodies. This is observed even in patients who have not been exposed to statins. The expression of HMG-CoA reductase is markedly increased when cells are exposed to statins and also in regenerating myocytes. Also, the premise is that MCH-I is upregulated, and statins induce an autoimmune myopathy [5]. All these suggest that statin-induced NAM is a distinct pathology from self-limiting statin-induced myopathy.

Being a rare entity, there are not many case reports that can differentiate the autoimmune response in patients taking hydrophilic versus lipophilic statins. Either way, as we could prove, even re-challenging patients with a “safer” type of statin does not have a benefit with regard to the muscular symptoms. Statins should be discontinued, and immunosuppressive therapy should be started to achieve amelioration of the patient's symptoms.

Treatment needs to be aggressive and initiated as soon as the diagnosis is confirmed. Steroids, methotrexate, and in severe cases IVIG can change the prognosis in this disease. IVIG therapy was initially introduced for the treatment of primary immunodeficiency diseases, but subsequently it has been used to target autoimmunity as well. The mechanism by which it exerts anti-inflammatory effects is still not fully understood, but it is known that the doses for treating inflammatory conditions are four to five times higher than for those used for replacement [13].

Administration of IVIG helps improving autoimmunity process by different mechanisms such as decreasing cytokines levels, blocking the inflammation, and decreasing IgG saturation, thus increasing IgG levels in circulation [13].

Only after one infusion of IVIG, our patient's symptoms have markedly improved and her CPK levels went back to normal, proving to be a successful approach.

## 5. Conclusion

Our case report reveals a rare entity, NAM often mistaken as statin-induced myalgia. The first instinct was to discontinue the offending drug to alleviate. Most often, this is the solution. However, after re-exposing the patient to a different statin that will cause recurrence of symptoms, we encourage the physicians to obtain a more detailed workup as that could change the prognosis of the patient's disease.

## Figures and Tables

**Table 1:** Myositis panel was negative except for SSA 52 Ab IgG which was equivocal.

Antibody	Result	Reference value
SSA 52 (Ro) Ab IgG	32 AU/mL	<29 AU/mL negative, 30–40 AU/mL equivocal, >41 AU/mL positive
SSA 60 (Ro) Ab IgG	4 AU/mL	<29 AU/mL negative
Ribonuclei protein U1 Ab IgG	0 AU/mL	<29 AU/mL negative
Jo-1(histidyl-tRNA synthetase) Ab, IgG	0 AU/mL	<29 AU/mL negative
PL-12(alanyl-tRNA synthetase) Ab	Negative	Negative
PL-7(threonyl-tRNA synthetase) Ab	Negative	Negative
EJ (glycyl-tRNA synthetase) Ab	Negative	Negative
OJ (isoleucyl-tRNA synthetase) Ab	Negative	Negative
SRP (signal recognition particle) Ab	Negative	Negative
Ku Ab	Negative	Negative
PM/SCL 100 Ab IgG	Negative	Negative
U2 sn (small nuclear) RNP Ab	Negative	Negative
Fibrillarin (U3 RNP) Ab, IgG	Negative	Negative
Mi-2 (nuclear helicase protein) Ab	Negative	Negative
P155/140 Ab	Negative	Negative
TIF-1 gamma (155 kDa) Ab	Negative	Negative
SAE1 (SUMO activating enzyme) antibody	Negative	Negative
MDA5 (CADM-140) Ab	Negative	Negative
NXP-2 (nuclear matrix protein-2) Ab	Negative	Negative
